# Recurrent Stress Cardiomyopathy: A Rare Variant in a Young Patient with Undiagnosed Pheochromocytoma

**DOI:** 10.1155/2021/5518578

**Published:** 2021-06-01

**Authors:** Robert Lembo, Paul Wesley, Joe B. Calkins

**Affiliations:** Department of Cardiovascular Medicine, Medical College of Georgia, 1120 15th Street, Augusta, GA 30912, USA

## Abstract

Biventricular stress cardiomyopathy is one of several known anatomical variants of reversible cardiomyopathies to occur. We present a case of a young patient with recurrent stress cardiomyopathy complicated by cardiogenic shock in the perioperative period. The cardiomyopathy observed was in a patient with neurofibromatosis type I and undiagnosed pheochromocytoma who presented for intervention of hydrocephalus. This case demonstrates the importance of vigilance in the young patient who develops shock in the perioperative period.

## 1. Introduction

Stress cardiomyopathy can often be encountered in the perioperative setting. Shock with rapid hemodynamic collapse in a young patient should raise concern for stress cardiomyopathy.

## 2. Case Report

A 28-year-old woman with a previous diagnosis of neurofibromatosis type I (NF I) presented to the hospital with dizziness, nausea, emesis, and anorexia. Magnetic resonance imaging of her head demonstrated a left thalamic mass and obstructive hydrocephalus. Seven years prior, the patient underwent ablation of atrioventricular nodal reentry tachycardia. Electrophysiology study at that time demonstrated dual AV nodal physiology, and she underwent successful cryoablation of her slow pathway without further history of palpitations. The ablation was complicated by biventricular failure requiring inotropic infusion for 12 hours. Rapid resolution occurred, and the patient was discharged without further event.

**Figure 1:**
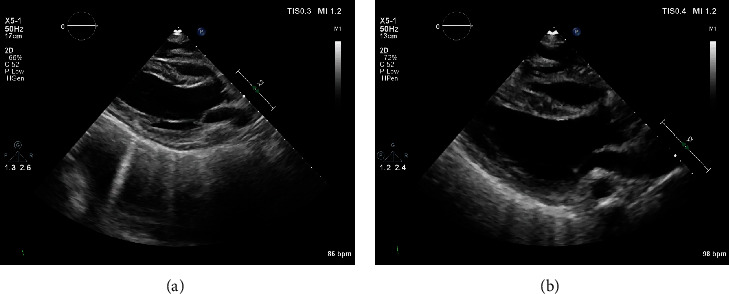
(a–b) Transthoracic echocardiogram demonstrating parasternal long axis view demonstrating hypokinesis of basal left ventricle and akinesis in all other segments (see supplementary material for video of echocardiogram clips in Figures 1–3). (b) Parasternal long axis view demonstrating recovery.

**Figure 2:**
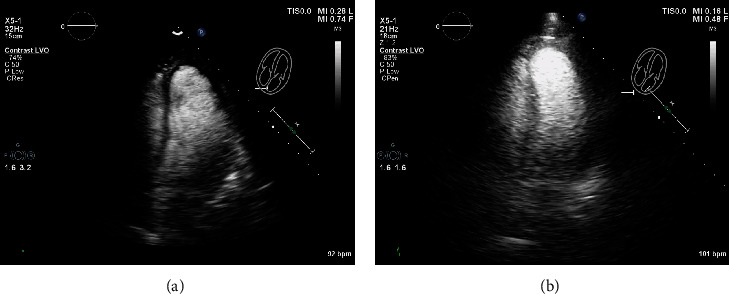
(a–b) Apical 4 chamber view with ultrasound-enhancing agent demonstrating hypokinesis of basal left ventricle and akinesis of all other segments. (b) Apical 4 chamber view with ultrasound-enhancing agent demonstrating recovery.

**Figure 3:**
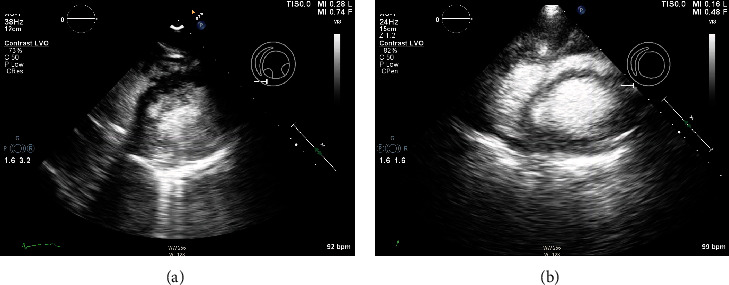
(a–b) Short axis view with ultrasound-enhancing agent demonstrating profound biventricular hypokinesis. (b) Short axis view with ultrasound-enhancing agent demonstrating recovery.

The patient underwent a biopsy of the left thalamic mass and drain placement to relieve obstruction. During anesthesia induction supraventricular tachycardia (SVT) occurred and was terminated with cardioversion. The procedure was aborted, and the patient was returned to the intensive care unit. A postoperative echocardiogram demonstrated severe left and right ventricular function and hypokinetic basal left ventricular segments, with akinesis of all other segments. The EF was less than 10% (see echocardiogram in Figures 1(a)–3(a)). Inotropic support with milrinone was initiated. Three days later, limited echocardiogram demonstrated normal left ventricular size with hyperdynamic systolic function and an EF greater than 70% (see echocardiogram in Figures 1(b)–3(b)). Metoprolol tartrate was initiated to prevent recurrence of SVT. The patient returned to the operating room and underwent successful external ventricular drain placement. Induction of anesthesia was uneventful. The patient was discharged without further cardiac event.

The patient again presented for symptoms with worsening hydrocephalus requiring further intervention. Upon anesthesia induction, the patient again developed SVT requiring cardioversion. Profound hypertension surrounded this arrhythmia with systolic blood pressure greater than 220 mmHg. Given the recurrent intraoperative hemodynamic events, testing of urinary and serum metanephrines was pursued which demonstrated elevated metanephrines and catecholamines. CT scan demonstrated a heterogeneous enhancing mass of the left adrenal gland and dotatate scan demonstrated increased uptake in the left adrenal gland suggestive of pheochromocytoma. Prazosin was initiated prior to definitive treatment of the hydrocephalus and pheochromocytoma. She underwent robotic-assisted laparoscopic left adrenalectomy in addition to left ventriculoperitoneal shunt placement. Pathology confirmed pheochromocytoma. The patient was discharged without further event and with normal left ventricular function.

## 3. Discussion

Pheochromocytoma and NF1 are rarely observed in the same patient and occur with an incidence of about 1% of individuals affected with NF1 [1]. Common presenting symptoms of pheochromocytoma are paroxysms of hypertension, headache, palpitations, and diaphoresis which occur in 50% of the cases. However, 10% of the patients remain normotensive and asymptomatic thereby limiting diagnosis [2]. It is common for pheochromocytoma to be first detected during anesthetic induction [3]. Uncontrolled catecholamine release can occur in the periinduction period causing cardiovascular complications including cardiogenic shock, takotsubo cardiomyopathy, myocardial infarction, and aortic dissection [4]. Furthermore, young patients without prior history who suffer from acute cardiac decompensation intraoperatively must be considered for entities such as pheochromocytoma. It has been suggested that supraventricular tachycardia in the presence of pheochromocytoma has led to acute depression in ventricular function, despite cardioversion of the arrhythmia [5].

As in this patient, the diagnosis of pheochromocytoma was considered after labile hemodynamics and rhythm abnormalities were encountered during two anesthetic inductions. Reversible biventricular dysfunction occurred after initial cryoablation seven years earlier, likely due to the same process; however, clinical details are limited. An atypical stress cardiomyopathy with biventricular dysfunction with hypokinesis at the base and subsequent akinesis in all other segments occurred in this patient.

Five anatomical variants of stress cardiomyopathy have been reported in the literature with apical ballooning (typical) occurring with a prevalence of 75-80% [6]. This biventricular anatomic variant that was observed in our patient occurs with a prevalence of less than 0.5%. Atypical patterns of left ventricular dysfunction are more common in young individuals and are associated with the development of shock. In the absence of LVOT obstruction, supportive care with inotropic use is indicated. Patients may require mechanical support in the form of an intraaortic balloon pump or percutaneous left ventricular assist devices until ventricular function recovers [5]. Kumar et al. report that a perioperative catecholamine surge may be attenuated with pretreatment with IV benzodiazepine, although this has not been extensively studied [3]. Recovery of left ventricular function from stress cardiomyopathy will vary from days to weeks as observed in the International Takotsubo Registry. Clinical factors unfavorable with ventricular recovery include male sex, baseline LVEF < 45%, and acute neurologic events associated with the cardiomyopathy [7].

This case demonstrates the importance of vigilance in the young patient who develops acute cardiomyopathy with biventricular involvement in the perioperative setting. Pheochromocytoma should be investigated in these instances. The biventricular variant of stress cardiomyopathy may be related to pheochromocytoma and appears to be reversible. Supportive care with inotropic therapy and serial echocardiographic evaluation is indicated and evaluation of possible pheochromocytoma is paramount.

## Additional Points

*Learning Objectives*. (i) Shock in the perioperative setting in young patients should raise the possibility of stress cardiomyopathy. (ii) Supportive care with inotropic therapy and serial reevaluation with echocardiography is paramount. (iii) Biventricular variant stress cardiomyopathy may be related to pheochromocytoma and appears to be reversible.

## Supplementary Materials

Supplementary 1 Transthoracic echocardiogram demonstrating parasternal long axis view (a) with hypokinesis of basal left ventricle and akinesis in all other segments with subsequent recovery (b).

Supplementary 2 Apical 4 chamber view (a) with ultrasound-enhancing agent demonstrating hypokinesis of basal left ventricle and akinesis of all other segments with subsequent recovery (b).

Supplementary 3 Short axis view (a) with ultrasound-enhancing agent demonstrating profound biventricular hypokinesis and subsequent recovery (b).
